# The Interaction of Bacteria with Engineered Nanostructured Polymeric Materials: A Review

**DOI:** 10.1155/2014/410423

**Published:** 2014-06-15

**Authors:** Ilaria Armentano, Carla Renata Arciola, Elena Fortunati, Davide Ferrari, Samantha Mattioli, Concetta Floriana Amoroso, Jessica Rizzo, Jose M. Kenny, Marcello Imbriani, Livia Visai

**Affiliations:** ^1^Materials Engineering Center, UdR INSTM, University of Perugia, 05100 Terni, Italy; ^2^Research Unit on Implant Infections, Rizzoli Orthopedic Institute, Via di Barbiano 1/10, 40136 Bologna, Italy; ^3^Department of Experimental, Diagnostic and Specialty Medicine (DIMES), University of Bologna, Via San Giacomo 14, 40126 Bologna, Italy; ^4^Department of Bioscience, University of Parma, Parco Area delle Scienze 11/a, 43124 Parma, Italy; ^5^Department of Occupational Medicine, Ergonomy and Disability, Nanotechnology Laboratory, Salvatore Maugeri Foundation, IRCCS, Via S. Boezio 28, 27100 Pavia, Italy; ^6^Department of Molecular Medicine, Center for Tissue Engineering (CIT), INSTM UdR of Pavia, University of Pavia, Viale Taramelli 3/b, 27100 Pavia, Italy; ^7^Institute of Polymer Science and Technology, CSIC Juan de la Cierva 3, 28006 Madrid, Spain; ^8^Department of Public Health, Experimental Medicine and Forensics, University of Pavia, Via Forlanini 2, 27100 Pavia, Italy

## Abstract

Bacterial infections are a leading cause of morbidity and mortality worldwide. In spite of great advances in biomaterials research and development, a significant proportion of medical devices undergo bacterial colonization and become the target of an implant-related infection. We present a review of the two major classes of antibacterial nanostructured materials: polymeric nanocomposites and surface-engineered materials. The paper describes antibacterial effects due to the induced material properties, along with the principles of bacterial adhesion and the biofilm formation process. Methods for antimicrobial modifications of polymers using a nanocomposite approach as well as surface modification procedures are surveyed and discussed, followed by a concise examination of techniques used in estimating bacteria/material interactions. Finally, we present an outline of future sceneries and perspectives on antibacterial applications of nanostructured materials to resist or counteract implant infections.

## 1. Introduction

There is an enormous need today for antimicrobial agents or coatings able to prevent material surfaces from colonization by microorganisms and periprosthetic tissues from infection. The increasing development of bacterial resistance to the most powerful antibiotics and the high rate of nosocomial infections are of great concern [1], since microbial contamination is a serious issue involving multiple spheres, including the health care and biomedical industries, water purification systems, and food packaging and storage. By way of example, each year about two million people acquire bacterial infections in American hospitals, with 90 000 yearly deaths [2]. Another pertinent fact is that almost half the people in developing countries suffer from water-related diseases, and more than three million people die annually from illnesses associated with unsafe drinking water due to the lack of disinfection. The European Antimicrobial Resistance Surveillance System (EARSS) reported the prevalence of methicillin-resistant* Staphylococcus aureus* (MRSA) in at least 10% of all* S. aureus*-associated infections in most European countries, rising as high as 40% to 50% in some cases, with a significant increase between 1999 and 2002 in Austria, Belgium, Germany, and the UK [3].

These alarming numbers underscore the importance of broad-range antimicrobial agents for infection prophylaxis in orthopedic trauma surgery. In response, numerous antimicrobial agents have been developed that can effectively inhibit the growth of microorganisms [4]. After the improvements achieved during the last few decades in terms of aseptic techniques, control of environment sterility, perioperative antibiotic prophylaxis, and anti-infective biomaterials have progressively become a primary strategy to prevent medical device-associated infections [5, 6]. The characteristics that antibacterial biomaterials should ideally possess are therefore diverse, while the required potency and spectrum of activity must reflect the risk rate and the etiology of infections under specific circumstances of use [7]. Furthermore, while antibacterial biomaterials are utilized for the construction of medical devices to be endowed with anti-infective adjunctive bioactive properties, ultimately determining their resistance to infections, they can also be employed to deliver medical substances, whose primary scope is the prevention, treatment, or reduction of infections, thus further widening the fields of application [8].

Hence, research concerning the development of antimicrobial polymers represents a great challenge for both the academic world and industry. Antimicrobial polymers can help to prevent biofilm development and to solve the problems associated with the use of conventional antimicrobial agents, such as residual toxicity, short-term antimicrobial activity, and development of resistant microorganisms.

Nanostructured materials with good antibacterial properties and biocompatibility/environmental safety have also been attained [7, 9–11]. Although the design and development of biomaterials have significantly advanced over the past decade, great challenges remain for fundamental exploration and practical applications.

With the advent of nanotechnology, an attempt was made to replace the biocides from antimicrobial paints with various nanosized substances such as zinc oxide, titanium dioxide, and silver [9, 12–14]. The application of nanotechnology concepts to medicine joins two large cross-disciplinary fields with an unprecedented societal and economical potential arising from the natural combination of specific achievements in the respective fields. The common basis evolves from the molecular-scale properties relevant to the two fields. The application of nanoscaled materials and structures, usually ranging from 1 to 100 nanometers (nm), is an emerging area of nanoscience and nanotechnology [15]. Nanomaterials often show unique and considerably changed physical, chemical, and biological properties compared to their macroscaled counterparts. They may provide solutions to technological and environmental challenges in the areas of solar energy conversion, catalysis, medicine, and water treatment [9]. Moreover, functional nanocomposites with desired properties can be tailored by incorporating specific nanomaterials and/or nanoparticles into the selected polymer matrix [10, 16, 17], while surfaces can be engineered by using different surface modification methods or coatings [9, 18].

**Figure 1:**
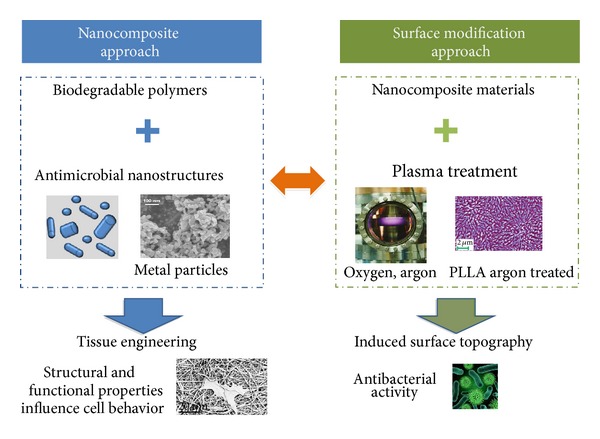
Schematic view of the two approaches applied in our laboratory to develop engineered nanostructured polymeric materials for antibacterial applications.

The aim of this paper is to bring attention to the evolution and potentiality of emergent active nanocomposite and surface modification approaches in antibacterial applications.  Figure 1 shows a schematic view of the two approaches used to develop engineered nanostructured polymeric materials for antibacterial applications: nanocomposites based on biodegradable polymers and antimicrobial nanostructures with potential application in tissue engineering and surface modifications based on plasma treatment in order to induce specific surface topography that affects the microbial vitality.

The focus of this review is on the relevant polymeric nanocomposite materials for antimicrobial applications (polymers, organic/inorganic nanostructures, and matrices) and the nanostructure interaction, including strategies for engineering surface in terms of modifications. Before introducing the antibacterial, nanostructured materials developed by using two different nanotechnological approaches, we will briefly review the mechanism of bacterial adhesion and biofilm formation on a surface. Finally, we will conclude by summarizing the techniques used in estimating bacteria-material interactions.

## 2. Bacterial Adhesion and Biofilm Formation

Tens of millions of medical devices are used each year. In spite of many advances in biomaterials, a significant proportion of all device types becomes colonized by bacteria and becomes the target of an implant-related infection [19]. Infection remains a major impediment to the long-term use of many implanted or intravascular devices and it is expected to further increase due to (i) the improved detection of biofilm-related infections by replacing diagnosis based on culture methods (often falsely negative) with reliable molecular techniques [20, 21]; (ii) the growing number of implanted devices in the aging population; and (iii) the increasing residency time of implanted devices, which are at continuous risk for infection during their implanted lifetime. Frequently, failure of implant devices stems from the bacterial colonization of polymer surfaces followed by the formation of a thick, multilayered biofilm which is extremely resistant to host defense mechanisms and antibiotic treatment. Often the only solution to an infected implanted device is its surgical removal.

**Figure 2:**
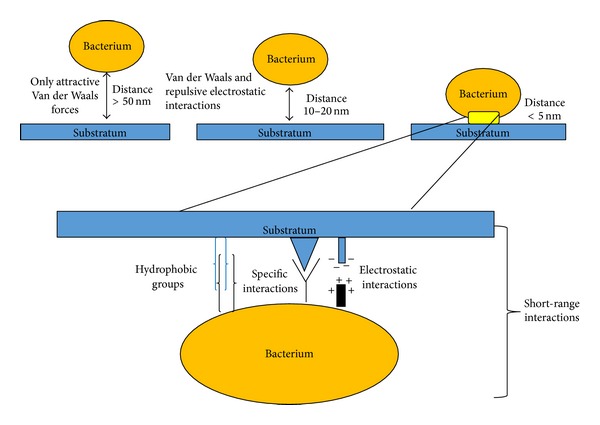
Phase one of bacterial adhesion consists in the initial attraction of the cells to the surface through the effects of physical forces. These physical interactions are further classified as long-range interactions (nonspecific, distances >50 nm between cells and surfaces) and short-range interactions (distances <5 nm, with involvement of hydrogen bonding, ionic and dipole interactions, and hydrophobic interactions).

Adhesion of bacteria to human tissue surfaces and implanted biomaterial surfaces is an important step in the pathogenesis of infection, whereby the bacteria can divide and colonize the surface [22–30]. Bacterial adhesion is an extremely complicated process that is affected by many factors, including environmental issues, the associated flow conditions, the presence of serum proteins or antibiotics, the bacterial properties, and the material surface characteristics [23, 31]. Bacterial adhesion to a material surface can be described as a two-phase process, including an initial, instantaneous, and reversible physical phase (phase one), followed by a time-dependent and irreversible molecular and cellular phase (phase two) [23, 31, 32]. Phase one of the bacterial adhesion consists in the initial attraction of the cells to the surface through the effects of physical forces, such as Brownian motion, van der Waals attraction forces, gravitational forces, the effect of surface electrostatic charge, and hydrophobic interactions [31–33]. These physical interactions are further classified as long-range interactions (nonspecific, distances >50 nm between cells and surfaces) and short-range interactions (distances <5 nm, with involvement of hydrogen bonding, ionic, and dipole interactions and hydrophobic interactions) (Figure 2). Long and short interactions are fundamental for the initial part of bacterial adhesion to surfaces, which makes the molecular or cellular phase of adhesion possible [23, 26, 31, 32]. The bacterial properties (bacterial hydrophobicity and bacterial surface charge) and the material surface characteristics (surface chemical composition, surface roughness, and surface configuration) are important in bacterial adhesion to uncoated surfaces and can potentially be targeted in antiadhesion therapy [33–35].

Phase two consists in molecular specific reactions between bacterial surface structures and substratum surfaces, uncoated or coated with host matrix proteins (i.e., albumin, fibronectin, fibrinogen, vitronectin, and laminin). To this end, the bacterial surface polymeric structures, which include capsules, fimbriae, or pili and slime, are responsible for the firm adhesion of bacteria to a surface. Attachment to materials coated with host matrix or plasma proteins is mediated by microbial surface components recognizing adhesive matrix molecules (MSCRAMMs) [36, 37].

**Figure 3:**
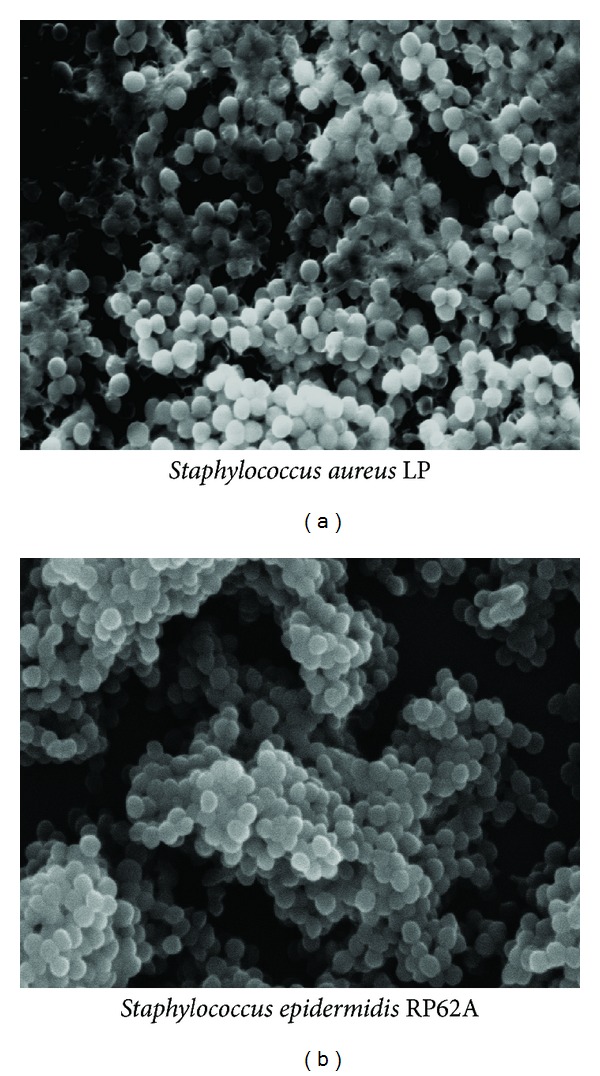
Scanning electron microscopy images of biofilm produced by* S. aureus* LP [116] and* S. epidermidis* RP62A [117] in the air liquid interphase of TSB-glucose medium after 24 h incubation at 37°C ((a) and (b): magnification, ×5 000).

Beyond phase two, certain bacterial strains are capable of forming a biofilm (Figure 3) if provided with an appropriate supply of nutrients. Bacteria frequently involved in biofilm-associated infections include the Gram-positive pathogens* Staphylococcus epidermidis*,* Staphylococcus aureus*, and* Streptococcus species* and the Gram-negative* Pseudomonas aeruginosa* and Enterobacteriaceae such as* Escherichia coli* [38].

**Figure 4:**
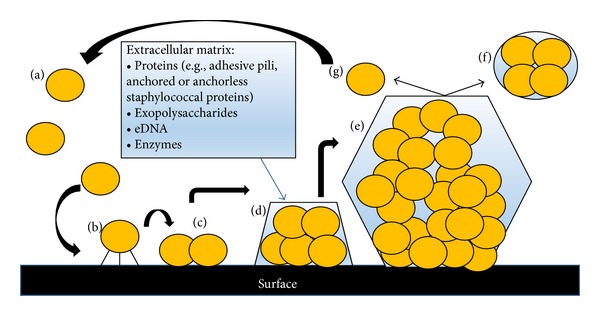
The different stages of biofilm formation: (a) planktonic cell; (b) reversible attachment to the surface; (c) irreversible attachment to the uncoated or protein coated surface; (d) formation of microcolonies through cell division and extracellular matrix production; (e) formation of a mature three-dimensional biofilm architecture showing pores for the passage of water. Cell detachment from the biofilm: (f) an active process leaving planktonic cell; (g) a passive process that can be shed through mechanical disruption adapted from [36].

Biofilm formation includes several sequential steps in which planktonic bacteria initially stick to a solid surface, which may be either unmodified or coated with host plasma proteins, followed by cell proliferation, cell-cell interaction, and production of an extracellular polymeric matrix, where bacteria accumulate in multilayered clusters (Figure 4). Following initial attachment, bacteria start growth and colonization, which results in the formation of contiguous cellular layers. The formation of multicellular clusters is based on intercellular adhesion and attachment of bacteria to a polymeric substrate produced, released, and integrated into the extracellular matrix. This step includes the contribution of the exopolysaccharides (e.g., staphylococcal polysaccharide intercellular adhesin (PIA),* P. aeruginosa* alginate), proteinaceous factors (e.g., adhesive pili, anchored or anchorless staphylococcal proteins), extracellular DNA (eDNA) [39], and enzymes. The subsequent development of the mature, three-dimensional biofilm architecture includes regulated motility. Once the structure has developed and matured, some bacteria detach and disperse into the surrounding medium, enabling the biofilm to spread over the surface [6, 30, 36]. Mature biofilms are highly resistant not only to the action of the innate and adaptive immune defense systems, but also to the action of antimicrobial agents and disinfectants. There are several possible mechanisms underlying this phenotypic resistance, which may depend both on the type of antibiotic treatment and the microorganism: slow rate of growth in the biofilm, altered metabolism, titration and inactivation of antimicrobial agents by the extracellular matrix, and the presence of an existing oxygen gradient that prevents the action of some antibiotics [40–42]. In addition, biofilms contain a large subpopulation of so-called persister cells that are constituted of dormant cells surviving antimicrobial treatment [43] and adapting to a slow growth rate through the emergence of small colony variants [41]. A limited diffusion of antimicrobials into biofilms has been suggested, but in most instances, no direct evidence has been provided [44, 45].

Biomaterial-associated infections have an enormous impact in terms of morbidity of the patients and costs to national health systems. In recent years, there has been increasing interest in anti-infective biomaterials aimed at counteracting the worrisome phenomenon of biomaterial-associated infections [46]. The creation of new anti-infective biomaterials can be obtained by alternative approaches oriented in different directions to promote a reduction in infection.

## 3. Polymeric Nanocomposite Approach to Provide an Antimicrobial Response

Polymer nanocomposites have attracted considerable attention in recent years and have become key materials in modern nanotechnologies [10, 17]. This interest arises because of their unprecedented performance, improved properties compared to the constituent parts, design flexibility, lower life-cycle costs, and uniquely large applicability of nanocomposites in various industrial fields. They consist of organic/inorganic nanoparticles incorporated in polymers that allow new materials to be obtained with modulated and distinct optical, electrical, and catalytic properties. These have potential applications in catalysis, bioengineering, photonics, and electronics [10, 17, 47].

In order to obtain an antibacterial polymeric nanocomposite, antimicrobial nanoparticles have to be selected and incorporated into the polymer matrices by conventional techniques (extrusion, injection molding, blow molding, etc.) [10, 17, 48–50]. Polymers are considered a good host material for metal nanoparticles; both biodegradable and nonbiodegradable polymer matrices are currently used in designing new nanocomposite systems with antimicrobial properties [10, 51].

Nanoparticles of noble metals have been studied with growing interest, since they exhibit markedly distinct physical, chemical, and biological properties from their bulk counterparts and there is a very strong interest in the use of metal and semiconductor clusters as advanced additives for plastics with considerable research done in this novel field of composite science. The metal and metal oxide nanomaterials commonly used as antimicrobial agents are silver (Ag), gold (Au), zinc oxide (ZnO), silica (SiO_2_), titanium dioxide (TiO_2_), alumina (Al_2_O_3_), and iron oxides (Fe_3_O_4_, Fe_2_O_3_) [7, 50–52]. Antimicrobial polymer additives are now available commercially. They are designed for various types of polymer matrices and processing techniques. These additives are often based on organic compounds or some metals [14, 51, 53–55]. However, it should be noted that only a marginal number of them are considered for medical use [56].

The chemical nature of the nanoparticles, their capping agent, and even the medium in which they are prepared might play an important role in determining the interaction between the polymer matrix and the nanofiller, thus affecting the dispersion and the bulk behavior of the nanocomposite films [57, 58]. Various methods to develop polymer nanocomposites by using metal nanoparticles have been developed, but sustained efforts need to be directed toward controllability for nanoparticle size, shape, distribution, and its interaction with polymers. Exploration of an inexpensive, easy fabrication method to fabricate polymeric nanocomposites with well-tuned nanoparticle size, shape, distribution, and interaction with polymers will be very important for various practical applications. Different chemical and physical methods exist to prepare metal polymer composites [54].

TiO_2_-chitosan nanocomposite is an optimal material possessing high potential for bone reconstruction, regeneration, and tissue engineering, based on its biomimic, bioactive, and biocompatible nature [48]. The smaller particle size of TiO_2_-chitosan increases the absorption of nutrients from the body and helps the formation of an apatite layer through the conventional principles of surface/volume ratio. Recently, TiO_2_-chitosan nanocomposites, synthetized with five different chitosan ratios, have proved to be promising biomaterials for orthopedic and tissue engineering applications. High surface area, appropriate hydroxyapatite formation, specific antibacterial action, increased cell viability, controlled swelling, and degrading rate are the favorably achieved features of the composite at a 2 : 1 titanium : chitosan ratio [48].

Another emerging class of nanoantimicrobials is bioactive copper (Cu) nanomaterials, which provide complementary effects and characteristics compared to other nanosized metals, such as silver or zinc oxide nanoparticles. It is known that copper is a broad-spectrum biocide and effectively inhibits the growth of bacteria, fungi, and algae. Recent studies have reported that nanoscale Cu exhibits good antibacterial activity [14, 59], and the products with Cu containing surfaces may meet hospital requirements [53]. Unlike silver (Ag), which has been studied extensively for antibacterial applications, Cu is an essential element for living organisms, and it might even be suitable for biomedical applications. Moreover, Cu is currently cheaper than Ag in the market and therefore, a method utilizing Cu would prove to be quite cost effective. The general mechanism of the antibacterial activity of nanoscale, metal-based materials is still uncertain. The antimicrobial activity of copper-based nanostructures depends on the microbial species and on the experimental setup.

Cu nanoparticle-coated cellulose films were developed through one-step reduction with antibacterial properties by [51]. The nanocomposite film exhibited strong, efficient antibacterial activity against* S. aureus* and* E. coli.* All the bacteria were killed within 1 h, and the dramatic reduction of viable bacteria could be observed within 0.5 h. The rapid killing effect was not only due to the release of Cu ions, but also due to contact killing. These characteristics increase the scope of applications of Cu/cellulose nanocomposite film in biomedical, catalysis, packaging, and electronics applications [51].

In the area of bioscience it was shown that square-patterned ZnO nanostructures integrated into biosensor arrays may allow ultrasensitive protein fluorescence detection, owing to the fluorescence-enhancement capability of nanoscale ZnO [60].

As an antibacterial agent, ZnO has several advantages: noticeable activity in the pH neutral region (pH = 7-8) without the presence of light [13]. ZnO nanoparticles were prepared within an alginate biopolymer by microwave (MW) treatment. The nanoparticles that were obtained were mostly spherical in shape and had a hexagonal crystal structure. The onset of the absorption of the ZnO-alginate nanocomposite solutions was shifted towards a lower wavelength due to the nanosized dimensions of the particles. A band-to-band recombination dominates the photoluminescence spectra of all the samples, while the intensity of the peaks that originate from the defects on the nanoparticle surfaces increases with the time of MW treatment. Antibacterial activity tests were carried out with* S. aureus* and* E. coli* pathogens. All the ZnO-alginate nanocomposite samples showed fast, strong antibacterial activity, with 99.9% reduction for* S. aureus* and 100% reduction for* E. coli* after 2 h of exposure [49].

Nanosilver is regarded as a new generation of antibacterial agents and the unique properties of silver nanoparticles (Ag NPs) have been extended into a broader range of applications. Incorporation of Ag NPs with other materials is an attractive method of increasing compatibility for specific applications [50]. Multifunctional nanocomposites based on biodegradable polymer matrix and silver nanoparticles have attracted great interest in nanobiotechnology due to the antimicrobial properties of silver, which are retained during polymer degradation [61, 62]. Silver species can be released in a controlled manner [63] and, for this reason, silver-containing materials have been extensively used to prevent the attack of a broad spectrum of microorganisms in different fields of application.

Dural Erem et al. [64] developed a series of PLA nanocomposite fibers containing, respectively, 0 wt%, 0.5 wt%, 1 wt%, 3 wt%, or 5 wt% Ag NPs, which exhibited increased antimicrobial activity, depending on the filler content. On the other hand, mechanical and thermal characterization tests, including thermogravimetric analysis, differential scanning calorimetry, and tensile testing, showed that increasing concentrations of silver hindered the mechanical properties of nanocomposites due to partial agglomeration, leading to the generation of flaws [64].

**Figure 5:**
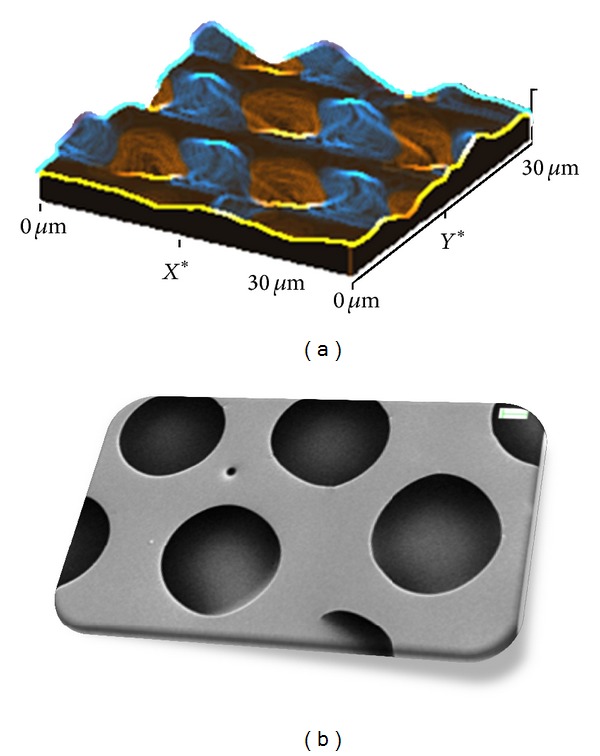
Atomic force microscopy (AFM) (a) and field emission scanning electron microscopy (FESEM) (b) images of PLGA/3Ag nanocomposite surfaces.

Our group previously reported the development of different Ag NP-based nanocomposites by using biodegradable polymers, in the form of poly(lactic acid) (PLA), poly(glycolic acid) (PGA) [11, 50, 57, 58, 65, 66], and poly(vinyl alcohol) (PVA) [16], and by means of different production processes, to be used in both biomedical and packaging applications. Nanocomposite films based on a biodegradable poly(DL-lactide-co-glycolide) copolymer (PLGA) and different kinds and concentrations of silver nanoparticles were developed by solvent casting. We demonstrated that PLGA film morphology can be modified by introducing a small percentage of silver nanoparticles that do not affect the degradation mechanism of the PLGA polymer in the nanocomposite.  Figure 5 shows atomic force microscopy (AFM) (a) and field emission scanning electron microscopy (FESEM) images of PLGA/Ag nanocomposite surface with 3 wt% of silver nanoparticles, developed by solvent casting process. Images underline the specific surface topography with the presence of a superficial, circular porous structure with a pore diameter of about 10 *μ*m. Results clearly evince the stabilizing effect of the Ag nanoparticles in the PLGA polymer and the mineralization process induced by the combined effect of silver and nanocomposite surface topography. The silver ion release can be controlled by the polymer degradation processes, showing a prolonged antibacterial effect [57, 58, 62, 65]. Results of the research suggest that the combination of biodegradable polymers and silver nanoparticles opens a new perspective for the use of nanomaterials with tunable properties in producing antimicrobial surfaces for biomedical applications.

**Figure 6:**
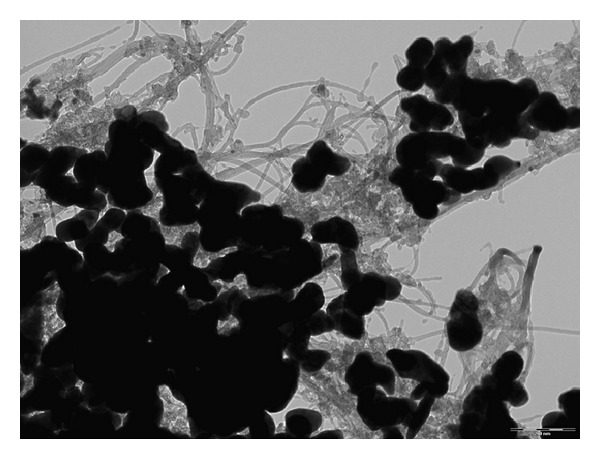
Transmission electron microscopy (TEM) image of poly(*ε*-caprolactone) (PCL) ternary nanocomposite based on SWCNTs and Ag NPs.

The antibacterial action of silver for biomedical devices has been the subject of numerous studies and, while opinions differ, the most probable scenario seems to be that silver ions bind to the bacterial cell membrane and damage it by interfering with membrane receptors and with bacterial electron transport. For this reason, we recently investigated the possibility of using a poly(*ε*-caprolactone) (PCL) biodegradable matrix reinforced with single-walled carbon nanotubes (SWCNTs) and silver nanoparticles as a potential support for primary human bone marrow-mesenchymal stem cells (hBM-MSCs) [67]. The newly designed SWCNT- and Ag NP-based materials possessed the unique properties offered by the synergistic interaction of the two different reinforcement phases.  Figure 6 illustrates transmission electron microscopy (TEM) images of the ternary nanocomposite, showing the interaction and the morphology of the two different nanostructures [65]. The suitability of these conductive nanocomposite films as support for hBM-MSC cells was demonstrated, showing comparable viability and cell/material interaction during the culture period. Moreover, it was proved that there is a clear concentration difference between the antimicrobial effects of nanosilver and potential adverse human cell or tissue reactions that encourage the application of the Ag NPs as conductive antimicrobial nanostructures able to induce stem cell activation.

The factors influencing bacterial adhesion to a biomaterial surface and, thus, the antimicrobial response include chemical composition [68], surface charge, hydrophobicity, and, in particular, surface roughness, topography, or physical configuration [26]. The electrospinning technique has proven to be a valuable method for developing antimicrobial PLGA nanocomposites based on Ag NPs [69]. Nirmala et al. reported on the electrospinning fabrication of cheap, stable, effective nanofiber mats with excellent antimicrobial activity, based on polyurethane (PU) nanofibers containing silver nanoparticles, that can be utilized to inhibit the microbial growth associated with food stuffs [70].

Recently in our laboratory we investigated the properties (mechanical, antibacterial, and degradation under composting conditions) of poly(lactic acid) and its composites prepared with a combination of microcrystalline cellulose (MCC) and silver nanoparticles, in order to show the prospective approach offered by these new multifunctional systems [66]. We demonstrated that the synergic effect of silver nanoparticles and cellulose structures increases the thermal and mechanical responses of PLA matrix. A bactericidal effect of Ag NP-based binary and ternary PLA nanocomposites on* S. aureus* and* E. coli* was detected at all time points and temperatures analyzed. The selected content of Ag NPs (1 wt%) in the nanocomposite formulations produced an evident antimicrobial effect and provided an active system for food packaging applications. Moreover, the low silver quantities do not influence the organic biowaste maturation process during the test for disintegrability under composting conditions. We have also found that the antibacterial activity of the PLA nanocomposites containing Ag NPs was greater on* E. coli* than on* S. aureus* cells, confirming previous work that explained that Ag nanoparticles appear more toxic to* E. coli* than to* S. aureus* [71].

In a recent study, melt-compounding extrusion followed by a film forming process was explored as a technique for preparing cellulose nanocrystal-based nanocomposites. High performance nanocomposites for packaging applications were produced by combining nanocrystalline cellulose (CNC) and silver nanoparticles with PLA polymer matrix; the antibacterial activity of these ternary systems against* S. aureus* and* E. coli* cells was studied [50]. For* S. aureus*, the bacterial activity was still remarkable in the presence of ternary systems, while the antibacterial effect of the nanocomposites was evident against the* E. coli* cells. The reduced antibacterial activity on* S. aureus* may be due to its structural character. Gram-positive and Gram-negative cells differ markedly in their cell walls. The thicker cell wall of* S. aureus* is of immense practical importance in protecting the cell from penetration of silver ions into the cytoplasm. The results of the study suggested that the better dispersion of Ag nanoparticles, confirmed by morphological, thermal, and mechanical analyses, positively affected the interaction of silver ions with the bacteria and this mechanism was found to be greater for* S. aureus* cells than for* E. coli*. This is due to different bacterial properties, suggesting perspectives for food packaging and hygiene applications that require an antibacterial effect that is constant over time.

## 4. Surface Engineering as a Strategy to Modulate Antimicrobial Response

The surface properties of biomaterials determine the kind and strength of communications between the biological environment and the materials. Recently D'Angelo et al. demonstrated that surface topography was able to induce stem cell differentiation as a single cue [72]. The factors influencing bacterial adherence to a biomaterial surface include chemical compositions [73, 74] surface charge [75], hydrophobicity [76], and surface roughness or physical configuration [77]. Depending on the hydrophobicity of both bacteria and material surfaces, bacteria differently adhere to substrates with modified superficial properties [78]. McAllister et al. found that the irregularities of polymeric surfaces promote bacterial adhesion [79, 80].

Modifying the surface characteristics of the biomaterial without altering the structural properties is, therefore, a strategy that has been used in recent years to obtain antibacterial materials [81]. The first essential step is the controlled fabrication of model surfaces.

This approach originates from the basic assumption that modifying the surface properties of a material (surface free energy, polarity, and topography) may result in diminishing bacterial adhesion during the initial stage of the biofilm formation process. For these reasons, many approaches to modify the surface of biodegradable polymer supports have been undertaken in order to introduce useful surface characteristics to the polymer. These can be mainly divided into (i) surface modification and (ii) surface deposition.

Surface modification can be performed either by applying wet chemistry through reaction with various chemical reagents or by applying high-energy electromagnetic radiation (e.g., by laser, ultraviolet radiation, and gamma rays). The interaction of a polymeric surface with electromagnetic radiation causes surface activation (through the breakage of accessible polymer bonds), permitting subsequent chemical modification [82–84]. Another promising method is modifying polymer surfaces by ionized gas (plasma). By using plasma processes, it is possible to change the surface chemical composition and properties such as wettability, surface energy, refractive index, hardness, chemical inertness, and biocompatibility [85]. This leads, naturally, to the selection of so-called cold plasma when the temperature of the treated material does not reach high values in comparison with the ambient temperature. This method demands low pressure (0.1–100 Pa) and the presence of a working gas (usually N_2_, O_2_, or Ar, CF_4_). Wan et al. demonstrated that appropriate oxygen plasma treatment could not only incorporate –C–O– groups onto the PLGA surface and increase its negative charges, but also produce peaks and valleys on its surface through an etching effect, thereby changing the surface topography [85].

**Figure 7:**
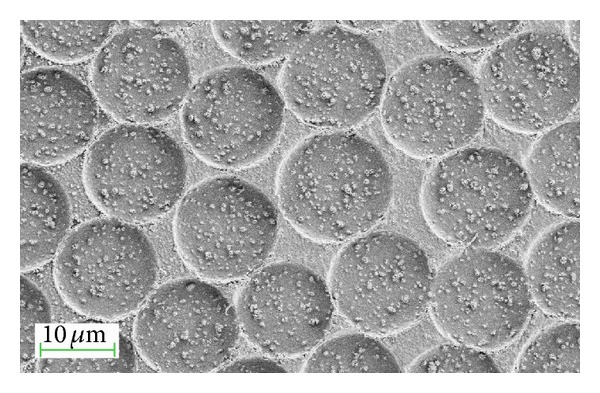
Field emission scanning electron microscopy image of the PLGA/3Ag nanocomposite film, modified by plasma oxygen treatment.

Since surface chemistry and surface topography are both important factors in influencing biological activity, we recently analyzed the combined outcomes of silver nanoparticles and radiofrequency plasma surface treatment on PLGA/Ag nanocomposite [11]. The study demonstrated that oxygen plasma surface treatment combined with a nanocomposite approach can readily reduce bacterial adhesion and growth on silver nanoparticles and PLGA systems. It must be noted that this reduction was shown for both types of tested bacterial strains (*E. coli* and* S. aureus*). The multistep approach we adopted showed itself to be a promising strategy to modulate the topographical and physicochemical surface properties of nanocomposite and, consequently, to regulate the antiadherence properties of biodegradable, PLGA-based systems by curbing the adhesion and growth of the two categories of tested bacteria [11].  Figure 7 shows a FESEM image of the oxygen plasma-treated PLGA/3Ag nanocomposite film developed by the solvent-casting method. The image shows the plasma effect that was aimed at changing the shape and depth of the initial pores and at inducing increased surface roughness, also demonstrating the etching effect of the oxygen plasma on the PLGA polymer. However, the plasma modifications to polymer surfaces are characterized by their weak stability over time, as polymer surfaces tend to return to their original chemical state [18, 86–92]. Another disadvantage lies in the complexity of the surface modification methods [11, 58].

An alternative strategy to prevent infection is by developing an antibacterial coating on the surface of the devices. Moreover, the application of silver nanoparticles on the surface of medical devices has been used to prevent bacterial adhesion and subsequent biofilm formation. The nanoparticles are either deposited directly on the device surface or applied in a polymeric surface coating.

Ho et al. reported the development of a long-term, active antimicrobial coating for surgical sutures. To this end, two water-insoluble polymeric nanocontainers based on hyperbranched polylysine (HPL), hydrophobically modified by using either glycidylhexadecyl ether or a mixture of stearoyl/palmitoyl chloride, were synthesized. Highly stabilized silver nanoparticles (Ag NPs, 2–5 nm in size) were generated by dissolving silver nitrate in the modified HPL solutions in toluene followed by reduction with L-ascorbic acid. Poly(glycolic acid)-based surgical sutures were dip-coated with the two different polymeric silver nanocomposites. The coated sutures showed high efficacies of more than 99.5% reduction of adhesion of living* S. aureus* cells onto the surface compared to the uncoated specimen. Silver release experiments were performed on the HPL-Ag NP modified sutures by washing them in phosphate buffered saline for a period of 30 days. These coatings showed a constant release of silver ions over more than 30 days. After this period of washing, the sutures retained their high efficacies against bacterial adhesion. Cytotoxicity tests using L929 mouse fibroblast cells showed that the materials are basically noncytotoxic [93].

Antimicrobial coatings have also been applied to venous catheters. Coatings containing combinations of antibiotics and antiseptics like minocycline and rifampin or chlorhexidine and silver-sulfadiazine have been applied to the internal and external surface of catheters. In several studies these antimicrobial-coated catheters were compared to noncoated catheters, and a reduction of catheter colonization and catheter-related bloodstream infections was found [94–96]. The antimicrobial effect of antibiotic-containing coatings was more pronounced than for the antiseptic coatings. Halton and Graves analyzed studies concerning economic aspects of catheter-related bloodstream infections and concluded that the use of antibiotic-coated catheters was clinically effective and cost-saving when compared to antiseptic-coated or standard catheters [97, 98].

## 5. Techniques Used in Estimating Bacteria/Material Interactions

In addition to the different types of nanostructured biomaterials that have been synthesized and developed, several experimental techniques have been developed to study and quantify bacterial adhesion and antibacterial activity on material surfaces [31, 32, 99–102].

Regarding bacterial adhesion techniques, the common element in all of them is that they measure* in vitro* the probability, force, or energy of attachment/detachment of many or single bacterial cells. Nevertheless, we need to point out that the* in vivo* adhesion process is complex and dynamic and these measurements might be misleading. These considerations may also apply to the* in vitro* determination of the antibacterial activity of biomaterials.

Regarding biofilm assessment, it is important to mention that several conditions during the biofilm formation process can affect the results, including growth conditions, the cultivation medium, and the surface selection. The exact size of the inoculums should be determined quite precisely by adjusting to a specific optical density or absorbance. Furthermore, the selection of medium composition for biofilm cultivation is crucial [103]. After the biofilm incubation step, other parameters need to be taken into consideration for biofilm quantification such as (i) the bacterial removal and rinsing procedures (3 washing steps with PBS); (ii) the measurement of planktonic growth before washing (normalizing biofilms formation by the growth index); (iii) the selection of the method for the target of quantification; and (iv) the interpretation of results and evaluation of assay quality [32, 102, 103].

Here, we summarize the* in vitro* techniques used in estimating bacteria-material interactions in static conditions: (a) bacterial/material adhesion and (b) antibacterial activity of materials.

### 5.1. Techniques Used in Determining Bacteria-Material Adhesion

To estimate bacterial adhesion, a previously prepared material surface is overlaid with a suspension of cells for a determined period and temperature of incubation. The size of inoculums/surface area, the time (1, 3 or 24 h), and temperature (4°, 22° or 37°C) of incubation may be very important parameters for bacterial adhesion quantification. Afterwards, the nonadherent cells are removed by rinsing or centrifugation and the remaining (adhered) cells on the surface are counted. When centrifugation is used to detach the nonadherent or weakly adherent bacteria, an overall estimation of the strength of adhesion may be calculated. The remaining (adhered) bacteria and biofilm can be examined by a number of methods [31, 32, 99, 100] (see (Table 1).

1. Viable bacterial counting methods include CFU plate counting, radiolabeling, 5-cyano-2,3-ditolyl tetrazolium chloride (CTC) staining, resazurin assay, and fluorescein diacetate (FDA) assay [32].
  - *CFU plate counting* is the most basic method for bacterial count. This technique is time consuming and involves tedious work using indirect and complicated procedures that give more uncertainty. Its great advantage lies in detecting only viable bacteria.
  - *Radiolabeling* is useful in the study of bacterial adhesion to irregular material surfaces. It is very sensitive and very accurate, allowing for rapid processing of a large number of samples. It requires special laboratory space and specific training for handling radioactive materials. Furthermore, a potential risk to researchers using radiolabeling techniques cannot be underestimated.
  - *CTC* is a tetrazolium salt and is reduced by this respiratory activity to form fluorescent CTC formazan on the cell surface. Therefore, CTC is used for specific staining of aerobic live bacteria and can be applied to hard-to-culture bacteria. However, since CTC alone is not sensitive enough to stain single cells, an enhancing reagent that improves the CTC staining efficiency has been added to commercially available kit.
  - *Resazurin assay:* resazurin, the main component of Alamar Blue, is a blue redox indicator that can be reduced by viable bacteria to pink resorufin; the extent of conversion from blue to pink is a reflection of cell viability. A calibration curve is necessary for data quantification.
  - *FDA assay* is based on the capability of viable microbial cells to convert noncolored, nonfluorescent, fluorescein diacetate (FDA) into yellow, highly fluorescent fluorescein by nonspecific intra- and extracellular esterases. A calibration curve is necessary for data quantification.
2. Microscopy for counting and morphological observation of adherent bacteria includes light microscopy, image-analyzed epifluorescence microscopy, scanning electron microscopy (SEM), confocal laser scanning microscopy (CLSM), atomic force microscopy (AFM), and Fourier transform infrared spectroscopy (FTIR) [32].
  - *Light microscopy* is a technique for bacterial counting and observation. Normally bacteria are stained with dyes like crystal violet or fuchsin. The advances in image analysis make bacterial counting by light microscopy much faster and more efficient. In this case, the substrata surfaces have to be translucent to be able to use light microscopy.
  - *Image-analyzed epifluorescence microscopy* allows live and dead bacterial cells on the surface to be distinguished if certain fluorochromes are used. Cell counting can be performed on an opaque surface. However, only two-dimensional imaging is possible and the use of fluorochromes is necessary for viewing bacteria. Furthermore, it is limited to macroscopic investigation of bacteria/surface interactions.
  - *SEM* is a well-established basic technique to observe the morphology of bacteria adhering to a material surface, the material surface morphology, and the relationships between the two. Environmental SEM and low vacuum SEM do not require metal or carbon sputtering and are less prone to damaging the bacteria adhering to a surface or to altering the surface characteristics of the specimen, thereby overcoming the previously mentioned drawbacks. However, SEM has some limitations: the enumeration of adhered bacteria is not feasible because of the small field and time-consuming work; it needs sample preparation for observation (tedious and labor-intensive) and the drying step is considered to cause noticeable cell shrinkage and other undesirable outcomes, like damage and distortion of the cell; it requires the specimen to be metal-sputtered; it cannot discriminate between live and dead bacterial cells; it also requires specialist equipment and specific training.
  - *CLSM* is a three-dimensional technique using fluorescent molecular probes and laser beams to study in situ bacterial associations with surfaces. It can be used to visualize and count bacterial cells directly on transparent or opaque surfaces. It allows the examination of the physiological state (live versus dead) of the adherent bacterial cells. This technique offers several advantages, including the ability to control depth of field, elimination, or reduction of background information away from the focal plane, and the capability to collect serial optical sections from thick specimens. The main disadvantages are the use of fluorescent probes to visualize bacteria and the high cost required for image quality.
  - *AFM* has been proved to be useful in imaging the morphology of individual microbial cells on solid surfaces, both in dried and hydrated states. It can be used for mapping interaction forces at microbial surfaces. AFM is a noninvasive microscopic technique capable of imaging surfaces at nanometer resolutions and three-dimensional images at high resolution. Furthermore, no sample staining, dehydration, or metallic coatings are necessary for this method. AFM image resolution is higher than that of environmental SEM. Nevertheless, there are some disadvantages such as the limitation of the observation area if compared with SEM and the unfeasibility of discriminating between live and dead bacterial cells. Furthermore, imaging bacterial cells can be a time-consuming task.
  - *FTIR* measures the vibrations of chemical bonds within all the biochemical constituents of cells (i.e., proteins, lipids, polysaccharides, and nucleic acids) and thus provides quantitative and qualitative information about the total biochemical composition of the intact whole microbial cell. The FTIR method is rapid, noninvasive, accurate, automated, inexpensive, and quantitative, allowing users to collect full spectra in a few seconds per sample.
3. Other direct and indirect methods include spectrophotometry, Coulter count, and biochemical markers (ATP) [32].
  - *Spectrophotometry* is a method measuring how much a chemical substance (i.e., bacterial cells) absorbs light as it passes through a sample. Unfortunately, it cannot discriminate between live and dead bacterial cells.
  - *The Coulter principle* is a versatile, robust, and accurate nonoptical method for counting and sizing particles of all types, including bacterial cells.
  - *ATP* is present in all cells and its determination is considered a valid biochemical marker of cell viability.
4. Molecular biological techniques: these techniques can be used to identify the total community of bacteria attached to a surface. They offer a very sensitive method for detection of specific genes or species by using polymerase chain reaction (PCR) and quantitative PCR (qPCR). One of the major limitations is related to the use of specific oligonucleotide probes that must bind specifically to the bacterial DNA sequence [32].
5. Determination of bacterial surface characteristics includes contact angle measurements. In the contact angle technique, a water droplet is applied to the surface of a dried lawn of bacteria. The angle formed where the water contacts the organisms is proportional to the surface hydrophobicity of the bacteria. Even if the analysis is very quick to perform, the contamination of test surface may alter the values obtained [32].
6. Methods for evaluating biofilm include biofilm recognition, thickness, and density measurements, and morphological observations can be performed by image-analyzed epifluorescence microscopy, SEM, CLSM, and AFM as previously indicated. Measurement of biofilm content can be determined by colorimetric biomass assay (crystal violet), Syto 9 assay (also used in CLSM studies of biofilm composition and morphology), resazurin assay, and FDA assay [32].
  - *Colorimetric biomass assay (crystal violet)*: crystal violet (CV) is a basic dye that stains both living and dead cells, by linking to negatively charged surface molecules and polysaccharides in the extracellular matrix. CV assay is economical and straightforward for all microorganisms. It cannot be used for cell viability because it cannot discriminate between live and dead cells.
  - *Syto 9 assay*: the fluorogenic dye Syto 9 is a nucleic acid stain, which diffuses passively through cellular membranes and binds to DNA of both viable and dead cells. As DNA is also a substantial part of the extracellular matrix, this staining will provide information on total biofilm biomass. Some limitations include no discrimination between live and dead cells; Syto 9, being a reagent, is quite expensive.

### 5.2. Techniques Used to Asses Antibacterial Material Properties

In recent years, nanostructured materials have attracted broad attention because of their novel physical, chemical, and biological properties as well as their potential use in many applications [9, 102, 103], especially those of metallic nanoparticles and their corresponding metal oxides, such as copper [14, 91, 104, 105], silver, [52, 62, 64, 93], zinc oxide, [13, 106], and titanium oxide [101, 107–109]. The antimicrobial activity of nanoparticles (NPs) has largely been studied with human pathogenic bacteria such as* Escherichia coli* [12, 105] and* Staphylococcus aureus* [12, 104]. The functional activities of NPs are influenced largely by the particle size, stability, and concentrations. NPs have been incorporated into polymeric nanocomposites using stabilizers or additives which allow them to be better distributed.

The strategy for testing the performance of anti-infective bioactive materials* in vitro* has to correspond to the characteristics of the test materials and their expected effects and mechanisms of action. Polycationic coatings or surfaces with immobilized antimicrobial substances that kill bacteria on contact should follow a different scheme of testing than disinfectant or antibiotic-releasing biomaterials. The same applies to the special case of biomaterial surfaces that act on bacterial physiology and biofilm structural integrity [102]. To estimate antibacterial material properties, a previously prepared material surface is overlaid with a suspension of cells for a determined period of time and temperature of incubation. The size of inoculums/surface area to test, the time (1, 3, and 24 h or more), and the temperature (4°C, 22°C, or 37°C) of incubation can be very important parameters for antibacterial activity quantification and are related to the material target application. It is important to note that, for the time-kill tests, a material can be defined as bactericidal only when abating the concentration of viable bacteria by at least 3 logs (99.9% when expressed in terms of percentage) [110, 111], while effects of lower order are not considered to be (clinically) significant. At the end of incubation, the bacterial suspension is removed from the surface and cell viability immediately examined by a number of different methods [102] (Table 2), as described below.

1. Evaluation of bacterial survivability: CFU plate counting, CTC staining, resazurin assay, and FDA assay. These methods are identical to the previously reported for bacterial adhesion quantification and can be used to evaluate bacterial cell survivability of the initial applied inoculum. The results must be compared with the material surface without the antibacterial substances or NPs.
2. Determination of antibacterial activity by agar well or paper disk diffusion method [112].
3. Determination of intercellular component leakage (cations, RNA, DNA, and protein): the leakage of intercellular components can be an indirect method for assessing bacterial membrane damage.
4. Microscopy for morphological observation: transmission electron microscopy (TEM), SEM, and AFM are used to show membrane damage and progressive destructions of cells directly in contact or after interaction with an antibacterial surface. Confocal laser scanning microscopy (CLSM) can be effectively performed to show cell membrane damage using the LIVE-DEAD BacLight bacterial viability kit. The kit includes two fluorescent nucleic acid stains: Syto 9, which penetrates both viable and nonviable bacteria and propidium iodide, which penetrates bacteria with damaged membranes and quenches Syto 9 fluorescence. Dead cells, which take up propidium iodide, fluoresce red, while cells fluorescing green are deemed viable.
5. Determination of lipid peroxidation products by spectroscopic studies: this type of determination has been reported especially with TiO_2_ surfaces. X-ray diffraction [113], laser kinetic spectroscopy, and attenuated total reflection Fourier transform infrared spectroscopy (FTIR) [114–117] have been used to show cell disruption due to lipid peroxidation or direct oxidation.
6. Evaluation of bacterial cell killing inside the biofilm is carried out by image-analyzed epifluorescence microscopy, SEM, CLSM, and AFM as well as colorimetric biomass assay (crystal violet), Syto 9 assay, resazurin assay, and FDA assay.

## 6. Conclusions and Future Research

Better understanding of the interaction between microorganisms, the implant, and the host may improve our current approach to the diagnosis and treatment of implant-associated infections [118–122]. Despite multiple efforts to discover medical therapies for treating biofilm infections, the physical removal of an infected medical device is often necessary, thus carrying an additional economic cost. There is consequently great interest in finding methods or strategies to inhibit biofilm formation [21]. Combined use of multiple antimicrobial agents with different chemistries and modes of action may be a strategy to improve the performance of these antimicrobial agents and circumvent bacterial adaptation [123]. However, the tremendous resistance of biofilms to conventional antibiotic therapy—together with the risk of a biofilm production induced by antibiotics themselves [124]—has prompted a great deal of research on synthetic surfaces and coatings that resist bacterial colonization. Several biomaterials used in orthopedic surgery demonstrate varying susceptibilities to infection because adhesion and growth of infecting bacteria are controlled by biomaterial surface properties like hydrophobicity [125] and roughness [125, 126]. Controlling the topography and hydrophobic properties of materials surfaces is thus a way to influence bacterial interaction with the surface and must be taken into account when developing novel, anti-infective biomaterials [127, 128].

However, since bacterial adhesion is a very complex process affected by many factors, such as bacterial and material properties and environment, further studies are required to understand the mechanisms of bacterial adhesion and implant infection and to provide adequate methodologies to prevent them from occurring. Future research must strive to better understand the pathogenesis of implant-related infections, with special attention to the alarming phenomenon of antibiotic resistance [129]. Future investigations should also focus on designing animal model systems to study* in vivo*-grown biofilms and infections.

The potential applications of nanotechnology for diagnosis, prevention, and treatment of diseases are currently very broad [129–132]. Nanostructured materials have been repeatedly shown to be able of improving biomaterial-cells interactions (e.g., osteointegration of bone implants or seeding of endothelial cells on vascular scaffolds) in comparison with the materials of the past [132–135]. Moreover, they appear endowed with the potential to contrast biomaterials infectability. Besides creativity and visionary power, practical application of nanomedicine requires simple approaches and systematic development. In this review, we have provided an overview on some fascinating developments in the area of nanomedical research and applications. Since the field is currently expanding at a very fast pace, we could not describe all aspects of current nanomedicine in detail. Our aim was mainly to give a view of developments and research topics in chemistry, biology, physics, and engineering that have the potential to revolutionize clinical therapies and diagnostics.

Surface engineering based on nanostructured materials offers a series of favorable features to contrast bacterial adhesion and biofilm growth. It also represents a valid alternative to classic antibiotic therapies or to antimicrobial-coated or -loaded biomaterials. Surface engineering, by acting on the nanotopology, reduces the area available for bacterial attachment or generates superhydrophobic surfaces. Nanostructured surfaces have been shown to be capable of altering the 3D conformation of adsorbed proteins and this could potentially have an effect also on host adhesins filming the biomaterial surfaces, thwarting the MSCRAMM-dependent bacterial adhesion. Many approaches of surfaces engineering are being proposed, all aimed at contrasting bacterial adhesion, each exhibiting some antiadhesive feature [7]. The identification of the most effective anti-infective solutions will require evidence-based data, obtained from multicenter clinical trials, together with appropriately designed and well-structured international registers. In the absence of these evidence-based data, even if myriads of new technologies will be introduced, the evaluation of antiadhesive nanoengineered biomaterial surfaces could run anyway the risk of remaining an uncertain matter as it is not robustly supported by reliable data [7].

## Figures and Tables

**Table 1:** Techniques used in determining bacteria-material adhesion.

**Viable bacteria counting methods**	**Microscopy for counting and morphological observation**	**Other direct and indirect methods**	**Molecular biological techniques**	**Determination of ** **bacterial surface characteristics**	**Methods of evaluating biofilms**
(i) CFU plate counting (ii) Radiolabelling (iii) CTC staining (iv) Resazurin assay (v) FDA assay	(i) Light microscopy (ii) Image-analyzed epifluorescence (iii) SEM (iv) CLSM (v) AFM (vi) FTIR	(i) Spectrophotometry (ii) Coulter count (iii) Biochemical markers		Contact angle measurements	Morphology: (i) Image-analyzed epifluorescence (ii) SEM (iii) CLSM (iv) AFM Biofilm content: (i) Crystal violet assay (ii) Syto 9 (iii) Resazurin (iv) FDA assay

**Table 2:** Techniques used in determining antibacterial activity of materials.

**Evaluation of bacterial survivability **	**Determination of antibacterial activity **	**Determination ** **of intercellular components leakage (cations, RNA, DNA, and proteins)**	**Microscopy for morphological observations**	**Determination of lipid peroxidation products by spectroscopic studies**	**Evaluationof bacteria killing throughout biofilm**
(i) CFU plate counting (ii) CTC staining (iii) Resazurin assay (iv) FDA assay	Well or paper disk diffusion methods	(i) Laser kinetic (ii) Spectrophotometry (iii) FTIR	(i) TEM (ii) SEM (iii) AFM (iv) CLSM		(i) Image-analyzed epifluorescence (ii) SEM (iii) CLSM (iv) AFM (v) Colorimetric biomass assays (crystal violet, Syto 9, resazurin assay, and FDA assay)
